# Tianshengyuan-1 (TSY-1) regulates cellular Telomerase activity by methylation of TERT promoter

**DOI:** 10.18632/oncotarget.13939

**Published:** 2016-12-15

**Authors:** Weibo Yu, Xiaotian Qin, Yusheng Jin, Yawei Li, Chintda Santiskulvong, Victor Vu, Gang Zeng, Zuofeng Zhang, Michelle Chow, Jianyu Rao

**Affiliations:** ^1^ Department of Pathology and Laboratory Medicine, David Geffen School of Medicine, University of California at Los Angeles, Los Angeles, CA, USA; ^2^ Beijing Boyuantaihe Biological Technology Co., Ltd., Beijing, China; ^3^ Genomics Core, Cedars-Sinai Medical Center, Los Angeles, CA, USA; ^4^ Department of Urology, David Geffen School of Medicine, University of California at Los Angeles, Los Angeles, CA, USA; ^5^ Jonsson Comprehensive Cancer Center, University of California at Los Angeles, Los Angeles, CA, USA; ^6^ Department of Epidemiology, School of Public Health, University of California at Los Angeles, Los Angeles, CA, USA

**Keywords:** TSY-1, hematopoietic cells, Telomerase, TERT, methylation

## Abstract

Telomere and Telomerase have recently been explored as anti-aging and anti-cancer drug targets with only limited success. Previously we showed that the Chinese herbal medicine Tianshengyuan-1 (TSY-1), an agent used to treat bone marrow deficiency, has a profound effect on stimulating Telomerase activity in hematopoietic cells. Here, the mechanism of TSY-1 on cellular Telomerase activity was further investigated using HL60, a promyelocytic leukemia cell line, normal peripheral blood mononuclear cells, and CD34+ hematopoietic stem cells derived from umbilical cord blood. TSY-1 increases Telomerase activity in normal peripheral blood mononuclear cells and CD34+ hematopoietic stem cells with innately low Telomerase activity but decreases Telomerase activity in HL60 cells with high intrinsic Telomerase activity, both in a dose-response manner. Gene profiling analysis identified Telomerase reverse transcriptase (TERT) as the potential target gene associated with the TSY-1 effect, which was verified by both RT-PCR and western blot analysis. The β-galactosidase reporter staining assay showed that the effect of TSY-1 on Telomerase activity correlates with cell senescence. TSY-1 induced hypomethylation within TERT core promoter in HL60 cells but induced hypermethylation within TERT core promoter in normal peripheral blood mononuclear cells and CD34+ hematopoietic stem cells. TSY-1 appears to affect the Telomerase activity in different cell lines differently and the effect is associated with TERT expression, possibly via the methylation of TERT promoter.

## INTRODUCTION

Over the past two decades, there has been intense interest in the study of the regulation of Telomerase, which is an RNA-dependent DNA polymerase. Telomerase contains a short RNA template complementary to the DNA Telomere sequence, as well as a catalytic protein with Telomerase reverse transcriptase activity. Telomerase is responsible for maintaining Telomere length. Earlier studies demonstrated that over 80% of all cancer types showed increased Telomerase activity and hence, increased Telomere length in cancer cells over their normal cell counterparts [1]. This together with activating oncogenes / deactivating tumor suppressor genes result in the dysregulation of tumor cell growth and proliferation [2, 3]. Due to its significant role in cancer biology, a considerable amount of effort has been devoted to developing specific inhibitors of Telomerase activities in order to develop targeted anti-cancer drugs. Thus far, these efforts have resulted in only limited success. This is partly due to the fact that cellular Telomerase homeostasis, including Telomerase activity and Telomere length, is regulated by a complex network of genes that have interconnected signals.

On the contrary, there have also been attempts to develop approaches to increase cellular Telomerase activity, as an auspicious anti-aging strategy [4, 5], or as a counteractive measure for some disease conditions such as bone marrow failure that are associated with decreased Telomerase activity. In fact, aplastic anemia (AA), the most severe form of bone marrow failure, is found to have a mutated telomerase gene resulting in the loss of Telomerase activity in hematopoietic cells in about one third of all cases. Even in those AA cases without the telomerase gene mutation and in cases of myelodysplastic syndrome (MDS), another form of severe bone marrow deficiency, decreased Telomerase activity in hematopoietic cells has also been seen [6–8].

Therefore, it seems that any efforts to target Telomerase activity, by either inhibition or stimulation, should be carefully tailored to an individual organ, system or cell type. Also agents that regulate the homeostasis of Telomerase activity may be superior to agents that drive the activity in only one direction, which may result in untoward side effects. In this study, we found that a Chinese herbal medicine, TSY-1, has a property that regulates Telomerase homeostasis. TSY-1 has been used to treat patients with AA for many years with some success, though definitive clinical trial data is not available at this time. Previously, two *in vivo* bone marrow deficiency mouse models, one induced with chemicals and the other induced with immune-suppression and radiation treatment, have both demonstrated strong bone marrow recovery after TSY-1 treatment. These studies appeared to reflect an effect of TSY-1 on modulating endogenous Telomerase activity [9–12]. Our current study demonstrated that TSY-1 has profound effects on maintaining the homeostasis of Telomerase activity: it inhibits the Telomerase activity in cancerous HL60 cells, which has high intrinsic Telomerase activity, whereas it increases Telomerase activity in normal peripheral blood mononuclear cells (PBMCs) and CD34+ hematopoietic stem cells (HSCs) derived from umbilical cord blood. Further, we demonstrated that such effects are associated with the epigenetic regulation of TERT promoter region.

## RESULTS

### TSY-1 inhibited Telomerase activity in HL60 cells but increased Telomerase activity in PBMCs and HSCs

**Figure 1:**
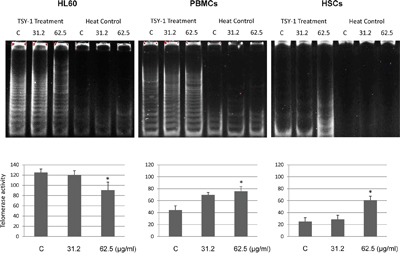
The changes of telomerase activity following TSY-1 treatment in HL60 cells, PBMCs and HSCs We detected telomerase activity using TRAP. Telomerase is a heat-sensitive enzyme. As a negative control, every sample extract to be evaluated was also tested for heat sensitivity. Thus, analysis of each sample consists of two assays: one with a test extract and one with a heat-treated test extract. Gel pictures showed that TSY-1 treatment decreased telomerase activity in HL60 cells but increased telomerase activity in PBMCs and HSCs. * P < 0.05, compared to untreated control, t-test. PBMC: normal peripheral blood mononuclear cells, HSCs: CD34+ hematopoietic stem cells, TRAP: PCR-based telomeric repeat amplification protocol.

To explore that TSY-1 has opposite effects on Telomerase activity between cancerous cells and normal cells, we chose human leukemia cells HL60, which was known to have high intrinsic Telomerase activity [12], and healthy donor-derived human PBMCs as well as core blood CD34+ HSCs with low intrinsic Telomerase activity. The effect of TSY-1 on Telomerase activity was tested using a quantitative telomeric repeat amplification PCR assay. Treatment cells were treated with 31.2 μg/ml and 62.5 μg/ml of TSY-1 drug for 24 hours and compared to the untreated control cells. As represented in Figure 1, we detected strong innate Telomerase activity in HL60 cells. With the treatment of TSY-1, the Telomerase activity decreased significantly in HL60 cells. However, TSY-1 treatment increased Telomerase activity in PBMCs and HSCs compared to the untreated control. Both of the changes (decreased Telomerase activity in HL60 and increased Telomerase activity in PBMCs and HSCs) showed a dose-response relationship. Taken together, these data confirm that TSY-1 has an opposite effect on leukemia cells with intrinsically high Telomerase activities (inhibitory) and normal blood mononuclear and stem cells with low Telomerase activities (stimulatory).

### TERT was identified as a target gene for TSY-1 activity

**Figure 2:**
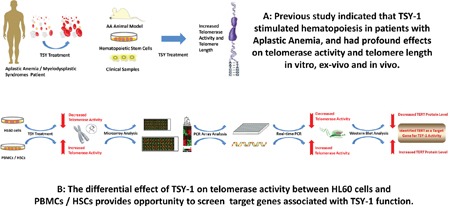
Diagram of strategies identifying target genes associated with TSY-1 activity

**Figure 3:**
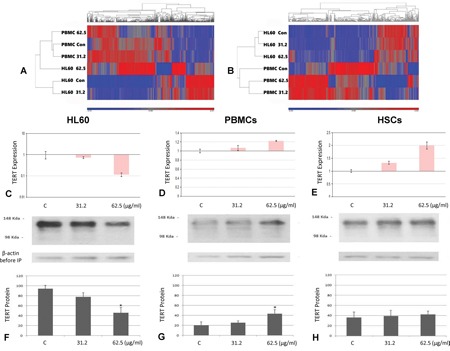
TERT gene and protein expression among different treatment and cells Heat map analysis of control versus TSY-1 treatment in HL60 cells (A) and PBMCs (B). **A.** up- and downregulation of the 765 genes that are differentially expressed between HL60 control and HL60 TSY-1 treatment. To generate the heat map, the data from all 6 samples is used to generate a standardized mean of 0 and a standard deviation of 1. Each sample is then compared to this mean to determine up or downregulation of the gene. The map shows that HL60 control and HL60 TSY-1 62.5 μg/ml are very different in gene expression. Note that PBMC control is very different (almost like opposites) compared to HL60 control. This indicates that a different set of genes are differentially expressed in the PBMCs. **B.** up- and downregulation of the 501 genes that are differentially expressed between PBMC control and PBMC TSY-1 treatment. The map shows the PBMC control and PBMC TSY-1 62.5 μg/ml are very different in gene expression. Note that PBMC control looks very different from HL60 control. **qPCR analysis of TERT gene expression in HL60 cells C., PBMCs D. and HSCs E.** TERT gene expression was measured by qPCR analysis. β-actin served as a reference gene for each triplicate set of reaction. Compared with the untreated control, TERT gene expression decreased in HL60 cells but increased in PBMCs and HSCs. * P < 0.05, compared to untreated control, t-test. **TERT protein expression in HL60 cells F., PBMCs G. and HSCs H.** The cells were treated by the TSY-1 of different concentration (31.2 μg/ml and 62.5 μg/ml). Cell lysates were immunoprecipitated with TERT antibody followed by SDS-PAGE analysis. Immunoblot picture shows TSY-1 treatment decreased TERT protein level in HL60 cells but not in PBMCs and HSCs. * P < 0.05, compared to untreated control, t-test. PBMC: normal peripheral blood mononuclear cells, HSCs: CD34+ hematopoietic stem cells.

The differential effect of TSY-1 on Telomerase activity in these cell models provided opportunities to identify potential target genes associated with TSY-1 activity (Figure 2). Using Affymetrix GeneChip Human Transcriptome Array system, we screened the entire genome in order to identify potential target genes that are associated with TSY-1-induced Telomerase modulation. The heat maps in Figure 3 demonstrate that both HL60 cells and PBMCs treated with TSY-1 62.5 μg/ml had a greater difference in gene expression compared to their untreated control cells. A total of 764 genes were differentially expressed between HL60 control cells and TSY-1 62.5 μg/ml treated cells, and 501 genes were differentially expressed between control PBMCs and TSY-1 62.5 μg/ml treated cells. TERT gene expression showed an opposite fold change after TSY treatment between HL60 cells and PBMCs (-1.82 VS 1.21), similar to the change pattern of Telomerase activity observed above. We then further identified genes that showed opposite expression patterns between HL60 cells versus PBMCs as well as HSCs, focusing on Telomere-associated genes. We performed a human Telomeres & Telomerase RT^2^ profiler™ PCR array analysis, which profiled the expression of 84 key genes central to Telomere replication, maintenance, regulation and Telomere-associated complexes. The results were shown in Table 1. The gene target that showed the most prominently differential effect between HL60 (down regulation) and PBMCs / HSCs (up regulation) is, again, the TERT gene.

**Table 1:** TSY-1 induced changes of expression in telomere associated genes in HL-60, PBMCs and HSCs

Telomere Maintenance				Telomere Associated Complex				Telomere Regulation				Other Telomerase-associated Genes			
Gene Target	HL60	PBMCs	HSCs	Gene Target	HL60	PBMCs	HSCs	Gene Target	HL60	PBMCs	HSCs	Gene Target	HL60	PBMCs	HSCs
DCLRE1C	−1.37	1.15	−1.33	RAP1A	1.11	−1.10	1.10	TGFB1	1.32	−1.08	1.12	ATM	−1.28	1.14	1.34
HSPA1L	4.52	5.74	2.85	ACD	−1.67	1.55	1.49	SSB	−1.76	1.04	1.17	CDK2	−2.59	1.06	1.00
MRE11A	−2.14	1.14	1.08	POT1	1.01	1.38	−1.32	SP1	1.07	1.05	−1.06	CHEK1	−1.73	1.83	−1.48
NBN	1.01	1..59	−1.05	TINF2	1.08	−1.01	−1.73	SART1	−1.24	1.02	1.37	CHEK2	−2.10	1.06	−1.12
PARP1	−2.22	1.10	−1.28	TERF1	−1.02	−1.57	−1.26	RB1	1.14	1.03	1.39	HAT1	−1.36	1.34	1.20
PIF1	1.36	1.69	2.05	TERF2	−1.13	1.11	1.08	RAPGEF1	1.25	−1.01	1.59	HNRNPA2B1	−2.08	1.11	1.03
PRKDC	−2.27	−1.15	−1.46	GAR1	−1.96	1.20	−1.47	RASSF1	1.96	1.23	1.05	HNRNPD	−1.47	1.21	1.08
PTGES3	−1.55	1.75	2.48	NHP2	−2.21	−1.21	−1.37	TP53	-	1.04	−1.14	HSP90AA1	−1.30	2.34	2.61
RAD50	−1.23	−1.12	1.05	NOP10	1.01	1.55	1.14	DCLRE1B	−1.76	1.01	−1.13	NCL	−2.08	1.05	−1.26
RFC1	−1.12	−1.05	−1.44	TERT	−5.55	1.69	8.12	KRIT1	−1.37	−1.07	−1.08	OBFC1	1.11	−1.60	−2.00
SMG6	1.11	−1.07	1.24	DKC1	−1.24	−1.17	−1.24	MYC	−4.58	−1.40	−1.32	PURA	1.64	1.31	2.35
TNKS2	−1.23	1.13	−1.06	EME1	−1.07	−1.89	1.13	SMAD3	1.25	1.69	8.12	RAD17	−1.13	1.05	1.19
RTEL1	−2.34	1.05	1.22	MSH2	−1.57	−1.17	−1.35	ABL1	−1.27	1.18	−1.24	RIF1	−1.30	1.11	−1.56
TEP1	1.08	1.50	1.29	MSH3	−1.52	1.07	−1.22	AKT1	−1.65	1.12	−1.08	SIRT2	−1.53	1.19	1.24
TNKS	1.03	−1.14	−1.15	MUS81	1.26	1.05	1.21	ATP5C1	−1.37	1.08	1.17	SIRT6	−1.34	1.13	1.38
POT1	1.01	1.38	−1.32	PLK1	−1.12	−1.75	−1.40	BCL2	−1.84	1.61	−1.28	SUN1	1.49	1.34	2.55
TINF2	1.08	−1.01	−1.73	SLX4	−1.06	−1.10	1.04	EGF	1.67	-	2.32	TP53BP1	−1.86	−1.08	−1.17
TERF1	−1.02	−1.57	−1.26	ERCC1	1.05	1.28	1.19	IGF1	-	-	4.51				
ERCC4	−1.05	1.05	1.16	ERCC4	−1.05	1.05	1.16	KRAS	−1.30	1.20	−1.07				
TERF2	−1.13	1.11	1.08	TERF2	−1.13	1.11	1.08	MEN1	−1.99	1.02	−1.20				
TERF2IP	−1.02	1.01	1.10	TERF2IP	−1.02	1.01	1.10	PAX8	−1.24	−1.26	2.48				
DKC1	−1.24	−1.17	−1.24					PINX1	−1.52	1.09	−1.03				
TERT*	−5.55	1.69	8.12					PPARG	7.30	−3.43	3.98				
MYC	−4.58	−1.40	−1.32					PPP2R1A	−1.20	1.28	1.20				
ERCC1	1.05	1.28	1.19					PPP2R1B	−1.42	1.41	−1.32				
ACD	−1.67	1.55	1.49					PRKCA	1.95	−1.43	1.91				
BLM	−1.97	1.17	1.13					PRKCB	1.93	1.00	1.21				

The gene target that shows the most prominent differential effect between HL-60 (down regulation) and PBMCs / HSCs (up regulation) is TERT gene. Others (ACD, BLM, ATP5C1, BLM, CDK2, ERCC4, HAT1, HNRNPD, HSP90A1, MRE11A, PPP2R1A, PTGES3, RAD17, RTEL1, SART1, and SSB), have the same direction, but with minimal effect (mostly <2-fold change). PBMC: normal peripheral blood mononuclear cells, HSCs: CD34+ hematopoietic stem cells.

To verify this result, a qPCR analysis was performed on TERT gene expression in three cell lines with and without TSY-1 treatment. As shown in Figure 3, our results confirmed that TSY-1 had an opposite effect on TERT gene expression between HL60 cells and PBMCs / HSCs. The 62.5 μg/ml concentration of TSY-1 drug had the maximum effect on TERT gene expression. Western blot combined with immunoprecipitation showed that TERT protein level in HL60 cells decreased significantly after TSY-1 treatment by a dose-dependent manner. In contrast, TERT protein level was elevated in treated PBMCs and HSCs compared with the untreated controls, although no significant difference existed between the control and treated HSCs (Figure 3). Therefore, in corroboration with the results from the Telomerase activity assays, it seems that the effect of TSY-1 on Telomerase activity may be mediated by TERT gene expression.

### TSY-1 treatment induced cell senescence in HL60 cells but not in PBMCs and HSCs

**Figure 4:**
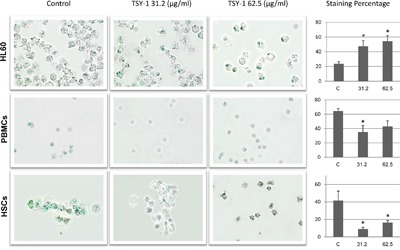
Effects of TSY-1 on beta-galactosidase labeling in HL60 cells, PBMCs and HSCs Cells were harvested and fixed, followed by cytospin and staining with senescence beta-galactosidase staining kit. Remarkably, the total number of healthy HL60 cells decreased after TSY-1 treatment, which did not happen in PBMCs and HSCs. The histogram shows the percentage of labeling cells following TSY-1 treatment increased in HL60 cells but decreased in PBMCs and HSCs. * P < 0.05, compared to untreated control, t-test. PBMC: normal peripheral blood mononuclear cells, HSCs: CD34+ hematopoietic stem cells.

Cell populations that stop growing can exhibit markers of senescence, including senescence-associated β-galactosidase staining. Cells stained dark blue are considered senescent cells, because the β-galactosidase activity was detected. Under the microscope field, we found that the total number of healthy HL60 cells significantly decreased after TSY-1 treatment, whereas the number of healthy PBMCs and HSCs increased. After treatment, the dark blue staining became larger and denser, and the percentage of labeling cells also increased in HL60 cells but not in PBMCs and HSCs (Figure 4).

### Demethylation / hypermethylation within TERT promoter positively related to gene expression after TSY-1 treatment

**Figure 5:**
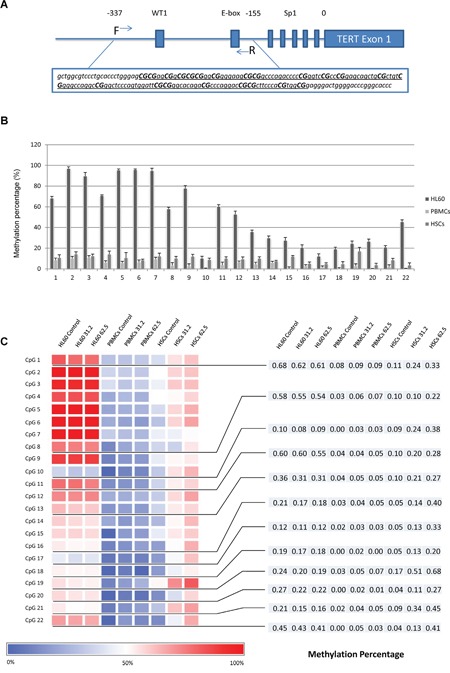
Distinct methylation profiles among different cells and treatment **A.** DNA fragment in TERT core promoter region was amplified. The underlined region was analyzed by pyrosequencing, which included 22 CpG sites. **B.** The percentage of methylation across 22 CpG sites among untreated HL60 cells, PBMCs and HSCs. **C.** Heatmap of DNA methylation profiling of TERT core promoter. The bottom blue-red scale bar refers to the degree of methylation measured by pyrosequencing. CpG 1, 8, 10, 11, 13, 16, 17, 18, 19, 20, 21 and 22 showed an opposite change of methylation pattern in a dose-response manner between HL60 cells (downregulated) and PBMCs / HSCs (upregulated). PBMC: normal peripheral blood mononuclear cells, HSCs: CD34+ hematopoietic stem cells.

Transcriptional regulation is a critical process to control TERT gene expression. In this study, the TERT gene expression changed within 24 hours, which underlines the possible role of transcriptional regulation. To confirm this, we focused on the epigenetic regulation within TERT core promoter region. Previous studies have chosen different regions of TERT promoter to explore the epigenetic regulation. Pettigrew et al. reported differential TERT promoter methylation and response to Decitabine in 20 CpG sites in acute myeloid leukemia cell lines [13]. We amplified a 185bp DNA fragment through bisulfite specific PCR. Then, through pyrosequencing, we analyzed the methylation patterns of 22 CpG sites in a region spanning 143bp (Figure 5A). Among the 3 untreated cell lines, the methylation levels of all 22 CpG sites in HL60 cells were remarkably higher than those of PBMCs and HSCs. The percentage of methylation in each CpG site gradually declined from upstream towards downstream (Figure 5B). Demethylation in HL60 cells (16 sites) and hypermethylation in PBMCs (17 sites) / HSCs (22 sites) that TSY-1 treatment induced were observed in a dose-response manner. In particular, 12 sites showed an opposite methylation pattern between HL60 cells (downregulated) and PBMCs / HSCs (upregulated), which were associated with concomitant decrease of TERT expression in HL60 cells and increase of TERT expression in PBMCs and HSCs (Figure 5C). Most of these sites were located in the hypomethylated downstream area. Among these 3 cell lines, we also observed that the most prominent differential effect of TSY-1 treatment on methylation was in HSCs.

## DISCUSSION

More than 80% of cancers have increased Telomerase activity [1], and Telomerase has served as an attractive target for anti-cancer drug development. Strategies including antisense oligonucleotide inhibitors, small molecular inhibitors, Telomerase-based immunotherapies and Telomerase-based oncolytic gene therapies have been explored [14, 15]. Clinical studies showed that when combining anti-Telomerase treatment with traditional therapies such as chemotherapy or radiotherapy, some success has been achieved. However, the overall benefit is rather limited. Part of the reasons may be due to the fact that Telomerase activity is rather dynamic and may be multi-directional during the course of cancer progression. It has been observed that shortened Telomeres not only limit the proliferative potential but also can be associated with increased tumor initiation, and promote chromosomal instability [16, 17]. This complex activating/repressive process makes it difficult to use Telomerase activator/inhibitor as a single target to regulate the cell growth and cancer development alone. Furthermore, as Telomerase inhibition may result in the inhibition of normal functions, especially hematopoietic stem cell functions, such a strategy may result in untoward side effects. Myelosuppression, anemia, neutropenia and thrombocytopenia have been commonly observed with the findings of recent clinical trials [18–20].

Likewise, because there are disease conditions that are associated with decreased or abnormal Telomerase function, some also attempt to develop approaches to increase Telomerase activity as an anti-aging strategy. Currently, the most studied method to extend Telomere is viral delivery [5]. Recently, Ramunas et al. delivered modified mRNA encoding TERT to human fibroblasts and myoblasts and found that this delivery could extend Telomeres rapidly and increase cell proliferation [21].

However, as exciting as the above target approaches may be, in theory, one may expect untoward side effects with such one directional approaches [22]. Application of Telomerase inhibitors in cancer patients may result in inhibition of bone marrow, whereas increased Telomerase activity may cause cell immortalization and promote carcinogenesis. The findings of increasing cancer risk from previous studies in animal models make Telomerase activators difficult to justify for their use in clinical practice [23–25].

TSY-1, a Chinese herb regimen, is rather unique. Our study shows that at least in hematopoietic cells, it has an effect of maintaining Telomerase homeostasis, i.e., inhibits Telomerase activity in intrinsically high expression malignant HL60 cells, whereas increases Telomerase activity in normal PBMCs and HSCs in a dose-response manner. Unlike most Chinese medicine, which is typically prepared by boiling, TSY-1 is extracted through a distillation process of multiple Chinese herbs: almond, pericarpium citri reticulatae viride, mangnolia officinalis, frankincense, and schisandra chinensis. Although it is difficult to identify a single effective constituent from multi-herbs, the standardized preparation can ensure consistencies with respect to concentration of active ingredients [26]. Recent studies utilizing mouse-models with both chemical-induced and radiation-induced bone marrow failure showed that the mice with bone marrow failure treated with TSY-1 had significant recovery of hematopoietic cells in both bone marrow and peripheral blood. In addition, TSY-1 induces extramedullary hematopoiesis in multiple organ systems including both liver and spleen and also specifically increases the number of CD4+/CD8- T cells and CD34+ cells (9, 10).

TSY-1 has been used in China for many years to treat AA and MDS. It is not until recently that studies have shown that bone marrow deficiency of MDS and AA are associated with Telomerase abnormality, whereas about one-third of congenital AA harbors mutations of the TERT gene resulted in loss of Telomerase activity [27]. In our previous study, we found that TSY-1 has the ability to recover bone marrow deficiency in *in vivo* animal model studies [12], and such an effect is associated with the Telomerase activity. Time course experiment on cultured cell lines showed the effect can be seen within 6-hour treatment. But we have not tested the effect of TSY-1 on Telomerase activity after removal of the TSY-1 treatment. It has been shown that a number of compounds can induce changes of Telomerase activity [28]. For example, hormones such as estrogen and testosterone and some growth factors increase the Telomerase activity, whereas differentiation agents such as retinoic acid have opposite effect. We previously tested granulocyte-macrophage colony-stimulating factor (GM-CSF), a growth factor stimulating hematopoietic cell proliferation and differentiation, which did not show effects on Telomerase activity when used alone (data not shown). Similar result was reported by others [29]. It should be noted that we used different batch of TSY-1 in the current study with the crude drug concentration of 0.62 g/ml, which was twice higher than what was used in the previous study (0.31 g/ml) [12]. Therefore, we used higher dilutions in the current study whereas the final effective concentrations of TSY-1 drug were similar.

By comparing global gene expression patterns of HL60, PBMCs and HSCs, we identified that the potential target gene of TSY-1 activity is the TERT gene. TERT is the major regulatory component of Telomerase activity [30]. Our results of TERT gene expression were consistent with the changes of Telomerase activity, which were further proved by the western blot analysis on TERT protein expression. Our β-galactosidase staining showed a distinctive change of cell senescence between HL60 and PBMCs / HSCs. More studies are still needed to determine whether these changes were directly modulated by TERT expression [31–34]. It is worth noting that we see a significant reduction of cell proliferation in HL60 cells but not in PBMCs and HSCs, further supporting the inhibiting effect of TSY-1 on human leukemia cells.

The observed modulating effect of TSY-1 on Telomerase activity in different cell systems suggests that TSY-1 may regulate Telomerase activity through epigenetic mechanisms, probably through TERT. Indeed, TERT core promoter contains abundant CpG sites. Several important transcription binding sites have also been identified [35–37]. E-box (located at -165 and +44 region) is considered as a binding site that can interact with Myc family oncogenes and may activate TERT transcription [38, 39]. GC boxes are other important binding sites for zinc finger transcription factor Sp1, which may contribute in maintaining the activity of the TERT promoter[40]. Generally, it is recognized that methylation of the CpG sites in the promoter region will inhibit transcription and result in gene silencing [41]. However, in terms of the TERT promoter, the examination of methylation status of CpG sites and TERT expression are rather controversial [42, 43]. Recently, studies indicated hypermethylation of the TERT promoter ubiquitously exists in highly proliferative cancer cells and correlates with increased Telomerase activity. On the contrary, many low proliferative normal cells demonstrate a hypomethylation in the TERT promoter region with decreased Telomerase activity [44].

We analyzed 22 CpG sites within the TERT core promoter region through pyrosequencing and found that Leukemia cell line HL60 demonstrated a significant hypermethylation within the TERT promoter compared to that of normal blood cells PBMCs and HSCs, supporting a positive relationship between hypermethylation in TERT promoter and Telomerase activity. After treatment, multiple CpG sites show changes of methylation percentage in parallel with TERT expression. This intriguing finding provides the foundation for the hypothesis that TSY-1 may exert the effect of maintaining homeostasis of Telomerase activity by epigenetic regulation. Yet more in-depth studies are still needed to elucidate exactly how TSY-1 affects the CpG island methylation. Further, it will be interesting to compare the effects of TSY-1 on TERT expression with other Telomerase inhibitors and activators, and also study the effect of TSY-1 on carcinogenesis.

In summary, TSY-1 appears to affect the Telomerase activity in different cell lines differently: inhibits Telomerase activity in intrinsically Telomerase-high leukemic HL60 cells but increases Telomerase activity in innately Telomerase-low normal PBMCs and HSCs. This effect appears to be mediated by methylation of the TERT promoter. The strategy of maintaining the homeostasis of Telomerase activity may have significant impact in developing approaches for Telomerase-based target treatment of bone marrow deficiency and cancer. Whether such an effect relates to the potential clinical benefit for patients remains to be tested in further studies.

## MATERIALS AND METHODS

### Cell culture, treatment and Telomerase activity assay

Human leukemia cells HL60 were obtained from American Type Culture Collection (ATCC, USA) and grown in Dulbecco's Modified Eagle's Medium (DMEM) containing 10% (v/v) fetal bovine serum (FBS) and 1% (v/v) streptomycin/penicillin (S/P). The human normal PBMCs and core blood derived CD34+ HSCs were isolated from healthy donors in the Center for AIDS Research virology core laboratory (University of California, Los Angeles). PBMCs were suspended in RPMI 1640 with 10% Human Serum AB. HSCs cells were grown in StemPro-34 Medium containing nutrient supplement, L-Glutamine (2mM), cytokines SCF (100 ng/mL), IL-3 (50 ng/mL) and GM-CSF (25 ng/mL). These cells were maintained at 37.0°C with 5% CO_2_. For all cell lines, medium was replaced every 2 to 3 days depending on cell density, which was maintained around 1 × 10^6^ viable cells/ml before TSY-1 treatment.

TSY-1 liquid of 10ml/vial, batch No. YZ130506, with a content of crude drug of 0.62 g/ml, with the purified drug concentration of 1mg/ml, was provided by Beijing Boyuantaihe Biological Technology Co., Ltd. The cultured cells were treated with 1/32 (31.2 μg/ml) and l/16 (62.5 μg/ml) dilutions of TSY-1 stock solution, which was determined by previous cell proliferation assay, for 24 hours prior to harvesting for the analysis of Telomerase activity and TERT expression. While the actual active ingredient(s) is not known at the moment, gas chromatography analysis has performed and identified a main peak that remains to be characterized. Cells cultured in medium treated with 0.01% Tween solution (the solvent of TSY-1 drug) were served as a TSY-1 untreated control.

Enzymatic activity was detected by the PCR-based telomeric repeat amplification protocol (TRAP), using the Quantitative Telomerase Detection Kit and following the manufacturer's instructions (US Biomax). As a negative control, every sample extract to be evaluated was also tested for heat sensitivity. Heat treatment was performed by incubating samples at 85°C for 10 minutes prior to the Telomerase activity assay. A dilution series of TSR control template was prepared in lysis buffer to serve as a standard curve. PCR was set at: 35 cycles of 94°C 30 s, 59°C 30 s and 72°C 30 s. The quantity of Telomerase substrate produced in each well was determined from a linear plot of the log_10_ of the quantities of the TSR control template standards versus the Ct values for their wells. Telomerase activity was determined as the relative ratios of the mean quantity of Telomerase substrate in each TSY treated sample to the mean quantity of Telomerase substrate in the untreated control sample. PCR products were also run on a 10% polyacrylamide gel in TBE stained with SYBR Gold Nucleic Acid Gel Stain.

### RNA extraction, gene expression analysis and quantitative PCR

Total RNA was extracted and purified from HL60, PBMCs and HSCs using an RNeasy Miniprep Kit (Qiagen). The effect of TSY-1 on gene expression profiles of HL60 cells and PBMCs was tested using the Affymetrix GeneChip® Human Transcriptome Array (HTA) 2.0 system. Total RNA samples were submitted to the UCLA Department of Pathology and Laboratory Medicine Clinical Microarray Core for processing. The Affymetrix WT PLUS Reagent Kit was used to prepare the RNA for hybridization to the HTA. Data analysis was performed using the Affymetrix Expression Console software and Transcriptome Analysis Console software. The Human Telomeres & Telomerase RT^2^ Profiler PCR Array was also used for the analysis of Telomere-associated gene expression (Qiagen).

TERT gene expression between control and TSY-1 treated samples was verified via qPCR using iTaq Universal SYBR Green Supermix (BIO-RAD) in iCycler iQ™ Real-Time PCR Detection System (BIO-RAD). Reverse transcription reaction was set at 42 °C for 90 min and inactivation at 85 °C for 5 min, followed by the cDNA amplification reaction: 95 °C 5min, 40 cycles of 95 °C for 30 s, 59 °C for 30 s and 72 °C for 45 s in 20μl reaction mixtures. Data acquisition was set at the chain extension step and the melt curve data collection analysis was performed between 55 and 95°C, with 0.5°C increments. Forward primer was 5'-GAGCTGACGTGGAAGATGAG-3' and reverse primer was 5'-CAGGATCTCCTCACGCAGAC-3'. Beta-actin was served as a reference gene for each triplicate set of reaction repeated three times. Results shown here are the mean normalized values of cDNA levels with the reference gene.

### Western blot analysis

Protein immunoprecipitation combined with Western blot was used to measure the expression of TERT protein. The control and treated cells were lysed in RIPA buffer. The extracts were incubated with human TERT antibody (Santa Cruz Biotechnology) and 20 μl of protein A/G Plus-Agarose (Santa Cruz Biotechnology). After washing and boiling, the samples were centrifuged to pellet the agarose beads, followed by SDS-PAGE analysis of the supernatant. To ensure use of equal amounts of protein per sample, blots were performed before immunoprecipitation experiments using Beta-actin. Cell protein extracts were resolved by SDS-PAGE (8%). The proteins were transferred onto a nitrocellulose membrane and immunoblotted with the primary antibody (1:2000) and secondary antibody (1:5000). After extensive washing, membranes were reacted with ECL detection reagents immediately prior to autoradiography. The relative levels of TERT protein were determined by scanning densitometry.

### DNA extraction, bisulfite treatment and methylation analysis

Genomic DNA was extracted from cells using the QIAamp® DNA Mini Kit (Qiagen). After the Nanodrop measured the concentration of DNA, the DNA stock solution was stored at -80°C until use. The extracted DNA samples were bisulfite and were treated using the EpiTect Bisulfite Kit (Qiagen) according to the manufacturer's protocol. The Human Methylated & Non-methylated DNA Set (Zymo) was used to assess the efficiency of bisulfite-mediated conversion of DNA.

To analyze the methylation in the TERT core promoter region, we performed PCR of bisulfite-converted DNA using the following primer pair: forward primer 5’-gttggcgtttttgtattttgg-3’, reverse primer 5’-gaatacccgaatccccaat-3’. For the purpose of pyrosequencing, the primers were HPLC purified and the end of the reverse primer was biotin labeled. The PCR reaction mixture is 50 μl in volume, containing 25 μl 2X iTaq™ universal SYBR^®^ Green supermix (BIO-RAD) with 200nM on each primer and 150ng of genomic DNA. PCR conditions used for the amplification were 95°C for 3 min; 50 cycles of 95°C for 15 s, 60°C for 30 s and 72°C for 30 s. 2 μl of PCR product was run on 2% agarose gel in TBE stained with SYBR Gold Nucleic Acid Gel Stain. The remaining product was used for pyrosequencing. The three sequencing primers were as follows: 5’-tttttgtattttgggag-3’, 5’-aagygyggtttagatt-3’, and 5’-ggtttttagtggatt-3’. The methylation percentage of each CpG site was presented and analyzed in a heat map.

### Senescence-associated β-galactosidase analysis

β-galactosidase stain was performed using a senescence β-galactosidase staining kit (Cell signaling Technology). All the cell lines were harvested and washed with 1×PBS, and were then fixed overnight at room temperature in fixative solution. We used cytospin to make cell slides and then immediately covered the cells by adding β-galactosidase staining solution on the slides. All the slides were incubated at 37°C (without CO_2_) overnight. Positively stained cells (dark blue) were counted in at least five microscopic fields with almost 500 counted cells. The ratio of the stained cells to the total number of cells was expressed as the percentage of senescent cells.

### Statistical analysis

Statistical analysis was performed using Microsoft Excel (Microsoft, Redmond, WA, USA) and SAS version 9.2 software (SAS Institute Inc., Cary, NC, USA.). Continuous data was presented as means plus/minus the standard deviation (±SD) and compared with the Student's t-test. Statistical significance was defined by a two-tailed p-value of 0.05.
